# Assessing Visuospatial Abilities in Healthy Aging: A Novel Visuomotor Task

**DOI:** 10.3389/fnagi.2016.00007

**Published:** 2016-02-01

**Authors:** Natalie de Bruin, Devon C. Bryant, Jessica N. MacLean, Claudia L. R. Gonzalez

**Affiliations:** The Brain in Action Laboratory, Department of Kinesiology and Physical Education, University of LethbridgeLethbridge, AB, Canada

**Keywords:** spatial ability, visuomotor, visuospatial, reach-to-grasp, sex, aging

## Abstract

This study examined the efficacy of a novel reaching-and-grasping task in determining visuospatial abilities across adulthood. The task required male and female young (18–25 years) and older adults (60–82 years) to replicate a series of complex models by locating and retrieving the appropriate building blocks from an array. The task allows visuospatial complexity to be manipulated independently from the visuomotor demands. Mental rotation and spatial visualization abilities were assessed. The results showed that the time taken to complete the tasks increased with increased mental rotation complexity. Patterns of hand use were also influenced by the complexity of the models being constructed with right hand use being greater for the less complex models. In addition, although older adults consistently performed the visuomotor tasks slower than the younger adults, their performance was comparable when expressed as the percent change in task demands. This is suggestive that spatial abilities are preserved in older adults. Given the ecologically validity, the described task is an excellent candidate for investigating: (1) developmental; (2) sex-based; and (3) pathology-based differences in spatial abilities in the visuomotor domain.

## Introduction

Spatial abilities are critical to functional independance. They allow us to locate targets in space, visually perceive objects, and understand the two- and three-dimensional (2D and 3D) spatial relationships among objects and our environment. These abilities allow us to safely navigate our environment through the accurate judgement of direction and distance. Spatial ability is not a unitary function, but rather it can be parsed into a number of distinct categories commonly classified as spatial visualization, spatial perception, and mental rotation. *Spatial visualization* has been defined as the ability to mentally manipulate complex spatial information when several steps are necessary for successful completion of a spatial task (Linn and Petersen, 1985; Voyer et al., 1995). An example of a task that could incorporate spatial visualization abilities would be arranging items so as to fit into a suitcase. *Spatial perception* is the ability to accurately establish spatial relationships with respect to one’s orientation despite the presence of distracting information (Linn and Petersen, 1985; Voyer et al., 1995). Spatial perception abilities are used when merging into moving traffic on a busy highway. The driver must determine whether the car will fit into the gap in the traffic while ignoring irrelevant surrounding vehicles on the highway. The third category of spatial ability, *mental rotation* is the ability to transform the orientation of a mental representation of an object in 2D or 3D space (Linn and Petersen, 1985; Voyer et al., 1995). Mental rotation abilities are used frequently throughout the day, for example, when combing one’s hair or applying one’s make-up in the mirror. There are numerous standardized spatial test batteries that have been developed to measure how participants solve spatial tasks. Examples of commonly applied tasks used to measure spatial visualization abilities are the Paper Form Board (Likert and Quasha, 1941), which requires participants to identify what an unfolded shape would look like once folded, and the Identical Block Test (Stafford, 1961) in which participants identify blocks from an array that match a reference block given a number of cues on the faces of the blocks. Two standardized tests that are used to assess spatial perception are the Rod-and-Frame Test (Witkin and Asch, 1948), which requires participants to identify horizontal or vertical lines presented in a rotated square frame and the Water Level Test (Piaget and Inhelder, 1956) in which participants indicate the orientation of the water line in the image of a tilted container. Lastly, numerous spatial tests have been developed to test mental rotation. The test that is most commonly used is the Mental Rotation Test (Vandenburg and Kuse, 1978) a variation of the original test developed by Shepard and Metzler (1971). This test requires participants to determine whether pairs of objects that have been rotated in depth relative to each other are identical or mirror images. Despite the clustering of spatial tests into the three general categories of *spatial visualization, spatial perception, and mental rotation* however, solving the tasks in a single test typically requires using multiple spatial processes. For example, tests assigned to the spatial visualization category (i.e., Paper Form Board task, Identical Block Test) likely include elements of mental rotation and spatial perception.

Our knowledge of how humans interact with their spatial environment has been largely based on studies that have used standard paper-and-pencil psychometric tests, computer-based chronometric tests (Linn and Petersen, 1985; Voyer et al., 1995), and more recently tests in immersive 3D environments (Parsons et al., 2004; Tsirlin et al., 2009). While these studies are critical to our understanding of spatial cognition, the low visuomotor requirements of the 2D tasks employed are often not representative of the physical interactions that we have with objects in our daily environment. Furthermore, given their complexity, many of the standardized tests are not suitable for use with young children, elderly, and patient populations.

We have developed a novel visuomotor task with variants that are appropriate for the range of spatial abilities from children as young as 3 years old (Sacrey et al., 2012) to old age (Gonzalez et al., 2014), as well as patient populations (unpublished). The task requires participants to locate, reach to, grasp, and manipulate the appropriate building blocks from an array of blocks to reproduce a 3D model. The task combines the three major categories of spatial abilities: mental rotation, spatial visualization, and spatial perception. Mental rotation abilities are challenged by determining whether 3D blocks in the workspace can be rotated to match the orientation of building blocks in the sample model irrespective of their orientation. In addition, spatial visualization abilities are used to identify the specific block that matches a building block in the sample model from an array of alternatives (that may differ by color, shape, and/or size; herein referred to as visuospatial search). Spatial perception abilities are also necessary for the majority of the task when the participant is identifying the correct building block among the array of distractors. This task, similar to the standardized paper-and-pencil and computer-based tests allows the level of visuospatial complexity to be manipulated while the visuomotor demands of the task are held constant. In contrast to these standardized tests however, the visuomotor demands of our task are extensive, matching the demands of everyday tasks. The developed task will allow the study of spatial cognition in the visuomotor domain, contributing valuable knowledge to our current understanding of spatial interactions in real-world scenarios.

The current study determined the feasibility of using a reach-to-grasp task to assess visuospatial and visuomotor function in male and female younger (18–25 years) and older (60–82 years) adults. To our knowledge, this is the first study to use a visuomotor task that combines aspects of spatial visualization (visuospatial search) and mental rotation. In this experiment, the visuospatial search demands were consistent but the spatial complexity of the models to be replicated was modulated across two conditions. In the low spatial complexity condition the position, the properties (i.e., color and size), and the orientation of each building block in the model to be replicated were visible from a single plane of view with the models having a “flat” configuration. In the high spatial complexity condition the model had a 3D configuration and needed to be rotated to ensure the accurate selection and placement of each building block in the model. The motor demands of the task (e.g., reaching to and grasping the blocks) were the same in both conditions.

The total time taken to replicate each model and the hand preference for each grasp was recorded. Given the reported decline in multiple measures of cognitive functioning with increasing age (Blanchard-Fields and Hess, 1996; Gabrowski and Mason, 2014), as well as the age-related deterioration observed in spatial visualization (Hertzog, 1989; Salthouse, 1990; Borella et al., 2014) and mental rotation (Willis and Schaie, 1989; Jansen and Heil, 2010; Borella et al., 2014) abilities we predicted an age-related decline in task performance. Furthermore, in accordance with the literature reporting superior performance for males compared to females on tests of mental rotation (McGlone and Davidson, 1973; Linn and Petersen, 1985; Voyer et al., 1995; Sherwin, 2003), we predicted that sex differences would emerge, with males consistently displaying a performance advantage.

## Materials and Methods

### Participants

Twenty-four self-declared right-handed young adults (YA; 12 males; 18–25 years) and 20 self-declared right-handed older adults (OA; 10 males; 60–81 years) were recruited from the university community to participate in this study. The study was performed with approval from the University of Lethbridge Human Subject Research Committee. All participants were naïve to the purpose of the study and provided written informed consent prior to the start of the study.

### Procedures

Participants were comfortably seated centrally in front of a table with a height of 0.74 m and a 0.70 m by 1.22 m workspace. Participants were instructed to replicate two series of four models. Subsequently, participants answered a modified version of the Edinburgh (Oldfield, 1971) and Waterloo (Brown et al., 2006) handedness questionnaires (see Stone et al., 2013 for complete description of modified questionnaire). Female older adult participants were asked whether they were using hormone replacement therapy in order to establish whether circulating sex hormone levels were likely to differ considerably within group.

**Figure 1:**
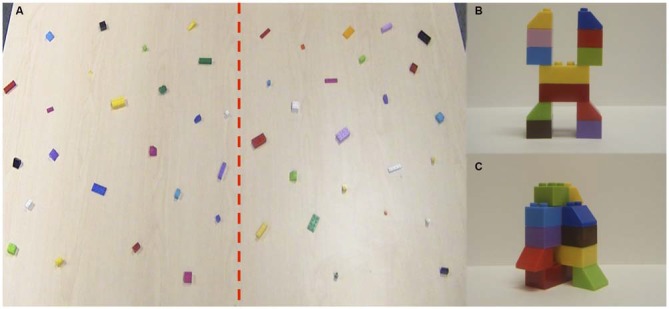
**Experimental set-up. (A)** Red dashed line divides workspace into right and left halves. Example of one of four **(B)** low (2D) and **(C)** high (3D) spatial complexity 12-piece model.

Forty-eight unique building blocks (LEGO^®^) were pseudorandomly distributed on the tabletop while participants were facing away from the table. A strip of clear tape was used to divide the workspace in half, and 24 blocks were distributed on the left and right sides (Figure 1A). Each trial began with the participants inspecting a 12-piece model they would replicate. Following inspection, the experimenter placed the model in the near right or left corner of the table (counterbalanced between trials). It has been shown that the position of the model on the table does not influence hand use (Stone et al., 2013). For each trial, participants were given the instructions to “replicate the model as quickly and as accurately as possible, using the pieces provided on the table.” No further instructions were given to the participants. Participants were free to manipulate and rotate the model to be replicated during construction. Following replication of the model, both models were removed and a different model to be replicated was provided. Building blocks were not replaced between trials. The same set of 48 unique building blocks was utilized for each set of four, 12-piece models in this experiment (Figure 1A). The two series of LEGO^®^ models differed with respect to their spatial complexity. In the low spatial demand condition (2D), the building blocks in the model to be replicated were in a “flat” configuration (Figure 1B). This allowed the participants to view the properties and orientation of all 12 building blocks from a single plane of view, which removed the need to physically rotate the model (although participants remained free to pick-up and manipulate the model to be replicated). In the high spatial demand condition (3D), the building blocks (the same as those used for the 2D models) in the model to be replicated were not all visible in the same plane (Figure 1C). This necessitated rotation of the model to allow accurate replication. Participants built four consecutive models in the 2D condition using all 48 blocks. Participants then built four models consecutively in the 3D condition, again using all 48 blocks. Start condition (2D, 3D) was counterbalanced and model presentation order was randomized between participants. The same eight models were used for all participants.

### Data Processing and Analysis

The total amount of time (i.e., latency, s) from the moment the participants lifted either hand from the table to initiate a reach towards the building blocks until the time when the replica model was placed on the table (inclusive of reaching, grasping, model manipulation, and model building) was recorded with a Tough Timer^®^ stop watch (Sportline Inc.). The task was recorded using a digital video camera (JV HD Everio^®^) placed directly in front of participants with a clear view of the workspace, building blocks, and participants’ hands. Each grasp was scored as a left- or right-handed grasp and right hand use was determined as a percentage of the total number of grasps for model construction (number of right hand grasps/total number of grasps × 100).

The effect of model Complexity and task progression on latency and hand use was compared between Sex and Group using mixed factor repeated-measures analyses of variance (RM ANOVA) with Complexity (2D, 3D) and Model (1–4) as the within-subjects factors and Sex (male, female) and Group (YA, OA) as between-subject factors. Subsequently, to allow the comparison of latency changes due to the mental rotation demands and not motor speed between younger and older adults the 3D latency data was normalized to the 2D data ([3D latency/2D latency]*100) and entered in a three-way RM ANOVA. Model number (1–4) was the within-subjects factor and Sex (male, female) and Group (YA, OA) were between-subjects factors. When statistical significance was determined the appropriate RM ANOVAs or paired *t*-tests were performed with bonferroni corrections for multiple comparisons used with the paired *t*-tests.

Data were analyzed using SPSS Statistics 18.0 for Windows (SPSS Inc., Chicago, IL, USA). Statistical significance was set at 0.05. Effect size (ES) was reported as η^2^ values.

## Results

All data were normally distributed and did not violate the assumptions of homogeneity of variance. Therefore, parametric statistics were used to analyze the behavioral data. Data are presented as means and standard deviations.

All participants self-reported as right-handers; this information was confirmed by the handedness questionnaire. Handedness scores differed between groups (*F*_(1,40)_ = 6.94, *p* = 0.012, ES = 0.148) with the OA reporting higher handedness score than the YA participants (YA = 30.5 ± 6.9; OA = 35.4 ± 4.7). This finding is consistent with previous reports (Gonzalez et al., 2014) that older participants tend to perceive themselves as more right-handed. Handedness scores were not differentially affected by Sex (*p* > 0.05). Age did not differ between sexes (*p* > 0.05). All female OA participants self-reported that they were not using hormone replacement therapy.

### Latency

#### Young and Older Adults

**Figure 2:**
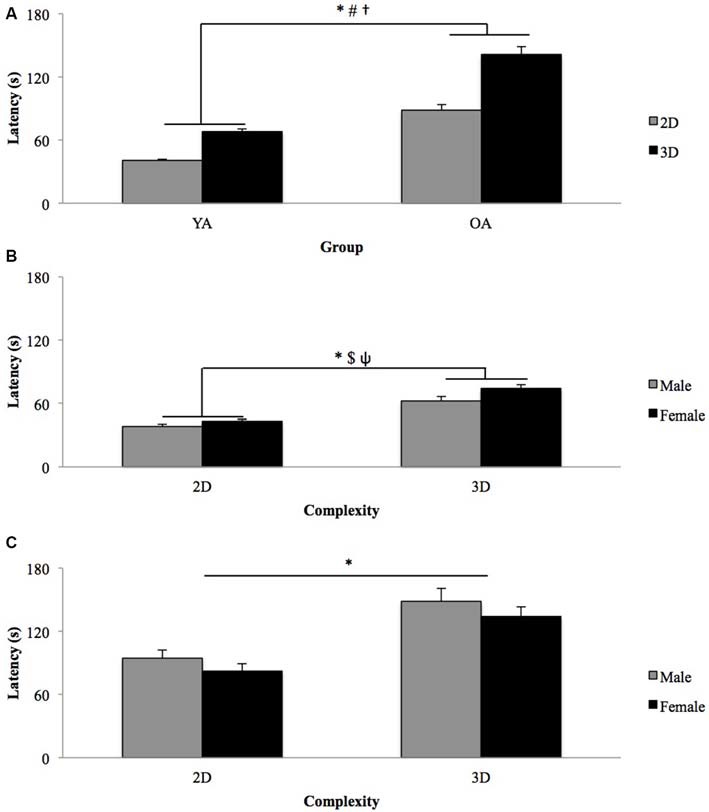
**Effect of Complexity of latency for (A) YA and OA, (B) male and female YA, and (C) male and female OA.** Data presented are means and standard errors. *Significant main effect of Complexity. ^#^Significant main effect of Group. ^†^Significant Complexity × Group interaction. *Significant main effect of Sex. ^Ψ^Significant Complexity × Sex interaction.

The analysis revealed a significant main effect of Complexity (*F*_(1,40)_ = 112, *p* < 0.001, ES = 0.737; Figure 2A), suggesting that participants constructed the 2D models significantly faster than the 3D models (2D = 62.4 ± 33.4 s, 3D = 101.5 ± 52.5 s). Latency was also affected by the order of model presentation (*F*_(3,120)_ = 19.0, *p* < 0.001, ES = 0.322) with earlier trials being completed more slowly than later trials (Model *1* = 97.8 ± 54.5 s, Model *4* = 69.7 ± 35.4 s), suggestive that the inherent visuospatial search associated with the task naturally declines with task progression as fewer blocks remain in the workplace and therefore fewer “distractor” blocks are present, allowing participants to identify the apposite block more readily. The Model by Group interaction also reached significance (*F*_(3,120)_ = 6.90, *p* < 0.001, ES = 0.147). *Post hoc* comparisons indicated that there was a significant decrease in latency for model construction from Model 1 to Model 3 for both groups, with YA (*t*_(23)_ = 4.77, *p* < 0.001) and OA (*t*_(19)_ = 4.74, *p* < 0.001) demonstrating a 8.8 s and 40.3 s decrease in latency respectively. Similarly, latency was significantly decreased from construction of Model 1 to Model 4 for both groups, with YA (*t*_(23)_ = 4.23, *p* < 0.001) demonstrating a 12.0 s decrease and OA (*t*_(19)_ = 4.39, *p* < 0.001)demonstrating a 47.4 s decrease. The Complexity by Model, and Complexity by Model by Group interactions were not significant (*p* > 0.05). A significant main effect of Group (*F*_(1,40)_ = 46.7, *p* < 0.001, ES = 0.539; Figure 2A) demonstrated that the YA completed trials significantly faster than the OA (YA = 54.5 ± 10.4 s, OA = 114.8 ± 41.5 s). The Complexity by Group interaction was also significant (*F*_(1,40)_ = 11.2, *p* = 0.002, ES = 0.220; Figure 2A). *Post hoc* pairwise comparisons suggested that there was an increase in latency from 2D to 3D model construction for both groups, with YA (*t*_(23)_ = 15.4, *p* < 0.001) and OA (*t*_(19)_ = 6.704, *p* < 0.001) demonstrating a 27.5 and 52.9 s increase in latency respectively. Sex did not differentially affect the average latency (*p* > 0.05).

Subsequently, to more closely examine the effect of model Complexity and task progression on latency separate three-way RM ANOVA were run for each group (YA, OA) in which Complexity (2D, 3D) and Model (1–4) were treated as within-subject factors and Sex (male, female) was a between-subject factor.

#### Young Adults

The analysis confirmed that YA completed the 2D models faster than the 3D models (*F*_(1,22)_ = 274, *p* < 0.001, ES = 0.926; 2D = 40.8 ± 7.4 s, 3D = 68.3 ± 14.2 s; Figure 2B). Latencies were also affected by the order of model presentation (*F*_(3,66)_ = 6.97, *p* < 0.001, ES = 0.241), with earlier trials being completed more slowly than later trials (Model *1* = 60.6 ± 14.2 s, Model *4* = 48.6 ± 13.3 s). A significant main effect of Sex (*F*_(1,22)_ = 4.38, *p* = 0.048, ES = 0.166; Figure 2B) revealed that male participants completed the task faster than the female participants (Males = 50.4 ± 10.5 s, Females = 58.7 ± 9.0 s). Lastly, a significant Complexity by Sex interaction (*F*_(1,22)_ = 4.75, *p* = 0.040, ES = 0.177; Figure 2B) suggested that the latency differed between male and female participants depending upon whether they were replicating the 2D or 3D models. *Post hoc* pairwise comparisons did not however reach significance (*p* > 0.05), with males constructing the models significantly faster than females in both Complexity conditions. Interestingly, when the YA participants were asked to complete a questionnaire regarding their comfort levels manipulating LEGO^®^ blocks it was found that the male and female participants had started playing with (*p* > 0.05; males = 4.2 years, females = 4.1 years) and had last used (*p* > 0.05; males = 13.4 years, females = 12.7 years) LEGO^®^ blocks at similar ages. Furthermore, when asked to indicate their comfort levels building with LEGO^®^ blocks (on a scale of one–ten where ten indicates “extremely comfortable”) there was not a significant difference between males and females (*p* > 0.05; male = 8.9, female = 8.2) suggestive that the male performance advantage was not simply a result of the male participants having greater experience with building LEGO^®^ models.

#### Older Adults

Similar to the YA participants, the OA completed the 2D models faster than the 3D models (*F*_(1,18)_ = 42.6, *p* < 0.001, ES = 0.703; 2D = 88.4 ± 33.9 s, 3D = 141.3 ± 54.0 s; Figure 2C). In addition, completion times were affected by the order of model presentation (*F*_(3,54)_ = 11.6, *p* < 0.001, ES = 0.392), with early models being constructed more slowly than later models (Model *1* = 142.4 ± 51.4 s, Model *4* = 95.0 ± 37.2 s). In contrast to the YA, however, latencies were consistent between sexes for the OA (*p* > 0.05). Furthermore, Sex did not differentially affect latencies by Model or Complexity (*p* > 0.05).

#### Percentage Change

**Figure 3:**
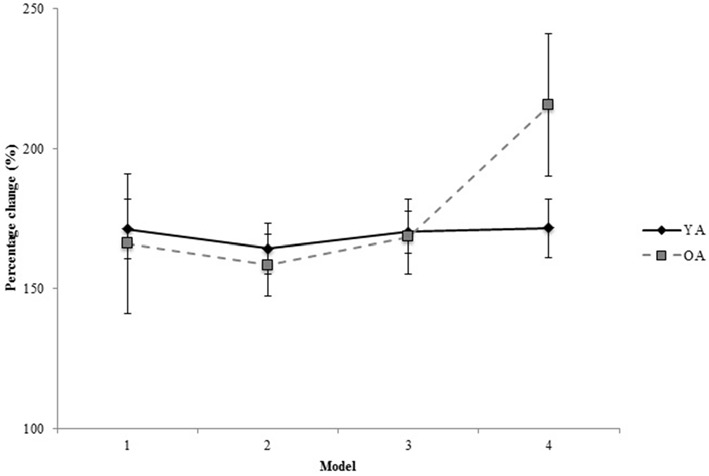
**Percentage change in latencies from 2D and 3D spatial complexity conditions for younger (YA) and older (OA) adults.** Data presented are means and standard errors.

When the data were normalized to further investigate the effects of the mental rotation demands of the task, the analysis did not reveal any significant main effects or interactions between factors (*p* > 0.05). In other words, YA and OA participants’ demonstrated comparable latency increase with increasing model complexity (YA = 167.4 ± 18.8%; OA = 163.9 ± 43.0%; Figure 3). This finding suggests that the spatial abilities required to complete this novel visuomotor task were similarly challenged in male and female participants, and that furthermore these spatial abilities appeared to be preserved with age.

### Hand Use

#### Young and Older Adults

The analysis revealed a significant main effect of Complexity (*F*_(1,40)_ = 5.12, *p* = 0.029, ES = 0.113) indicating that participants used their right-hand more during construction of the 2D models when compared to the 3D models (2D = 75.5 ± 15.5%, 3D = 72.0 ± 15.1%). Hand use was also influenced by the order of model presentation (*F*_(3,120)_ = 12.4, *p* < 0.001, ES = 0.236) with participants’ right hand use varying between 80 and 68% between construction of Model 1 and Model 4 (Model *1* = 80.2 ± 15.6%, Model *2* = 68.0 ± 19.7%, Model *3* = 76.4 ± 18.1%, Model *4* = 70.4 ± 18.0%). The Model by Group interaction was also significant (*F*_(3,120)_ = 38.0, *p* < 0.001, ES = 0.386). *Post hoc* pairwise comparisons did not reach significance for the YA group. The OA group, however, used their right hand significantly more when constructing Model 1 when compared to: Model 2 (*t*_(20)_ = 5.02, *p* < 0.001; Model *1* = 88.2 ± 12.8%, Model *2* = 66.0 ± 21.3%) and Model 4 (*t*_(20)_ = 4.18, *p* = 0.001; Model *4* = 71.0 ± 20.6%). They also used their right hand significantly more when constructing Model 3 when compared to: Model 2 (*t*_(20)_ = 5.00, *p* < 0.001; Model *3* = 86.9 ± 15.1%) and Model 4 (*t*_(20)_ = 4.179, *p* = 0.001). Group and Sex did not differentially affect average right hand use (*p* > 0.05).

## Discussion

This study developed a novel assessment tool for visuospatial abilities in the visuomotor domain. To our knowledge, this is the first study to describe and assess an interactive visuomotor task that challenges both spatial visualization and mental rotation abilities. The task required that participants replicate complex models by locating and selecting building blocks that vary in characteristics such as shape, color, and size from an array of blocks. The study found that the time to complete each model decreased in both spatial complexity conditions with the construction of consecutive models for both participant groups. This decrease in time suggests that the visuospatial search requirements of the task naturally diminished as blocks (and therefore “distractors”) were removed from the workspace and incorporated into the models. Confirming that the spatial complexity of the models was different between conditions, both groups of participants took longer to complete the models in the more spatially complex (3D) condition. Because the 2D and 3D models were comprised of the same number of identical blocks the difference in time likely reflects the increased spatial complexity of the 3D models. Moreover, because the manipulation was in the dimensional composition of the models, the time difference between conditions persisted through all four models.

An important finding of the current investigation was that spatial abilities are preserved in the OA participants. Compared to YA, OA participants displayed slower trial times across experimental conditions. This is likely due to differences in familiarity with the task (one might argue that young adults have had more experience “playing” with LEGO than older adults) and in age-related decline of perceptual and motor speed (e.g., Goggin and Meeuwsen, 1992; Chaput and Proteau, 1996). However, when data was normalized and expressed as percentage of the less demanding visuospatial task (2D models) YA and OA participants behaved similarly. In other words, the proportional increase in task completion times from the low to high visuospatial complexity conditions did not differ between the YA and OA, suggestive that the specific visuospatial abilities challenged by the developed task are in fact preserved in older age. This is an important finding because it remains unclear which visuospatial processes are affected by age and which are spared (for a review, see Iachini et al., 2009; Klencklen et al., 2012). For instance, some studies have shown an age-related decline in the ability to mentally rotate visual images, in the ability to retrieve spatiotemporal sequences, and in visuospatial imagery (Berg et al., 1982; Craik and Dirkx, 1992; Iachini et al., 2005; Ruggiero et al., 2008). Other studies have shown preserved spatial abilities in the elderly (Cherry and Park, 1993; Parkin et al., 1995; Yamamoto and Degirolamo, 2012). For example, Yamamoto and Degirolamo (2012) asked young and senior participants to learn landmark locations in virtual environments either by navigating in them in the first-person perspective or by seeing aerial views of the environments. Spatial learning performance was less accurate for the seniors when navigating in the first-person but equally accurate to the young adults when navigating using the aerial view. These studies and the results of the current investigation strongly suggest that the consequences of aging in spatial cognition are different depending on the type of spatial process that is challenged. Because the task used in the present study resembles everyday actions (i.e., reaching and grasping for objects), the current investigation also contributes to the evidence showing a less steep (or an absence of a) decline in spatial abilities in familiar ecologically relevant spatial tasks when compared to abstract laboratory tests (De Beni et al., 2006; Iachini et al., 2009).

Several studies have shown that males perform better in tasks that involve mental rotation, 3D figures, and spatial perception (McGlone and Davidson, 1973; Linn and Petersen, 1985; Voyer et al., 1995; Sherwin, 2003). The different levels of visuospatial complexity used in the present tasks were sufficient to produce the sex differences which had previously been assessed by paper-and-pencil tests and computer-based chromomeric tests (e.g., Linn and Petersen, 1985; Voyer et al., 1995; Sherwin, 2003). In the current experiment, the young male participants performed the tasks significantly faster than young female participants. Puzzling, the sex difference present in YA participants was not observed in OA. This was unexpected, as some studies have reported that sex-related performance differences in visuospatial tasks are present in the elderly (Berg et al., 1982; Willis and Schaie, 1989; Jansen and Heil, 2010). The studies reporting the presence of performance related sex differences in older adults have however, utilized standard paper-and pencil tests. In contrast, the developed task required participant’s to interact with the stimuli, to mentally rotate building blocks prior to grasping, and to orient the block appropriately in order to add it to the 3D model being assembled. It is probable that the novel interactive nature of our task is responsible for the inconsistence between our study and previous studies that have assessed spatial abilities in OA. While it also remains possible that the noted sex differences are a result of the young male participants having more experience “playing” with building blocks than the young female participants, a difference that would likely dissipate with age, this appears unlikely to be the overriding contributing factor. When the YA were presented with a questionnaire regarding their comfort manipulating LEGO^®^ blocks there were no reported differences between the responses of the young male and female participants. It is also possible to speculate that sex-steroid levels which have been theorized to contribute to sex differences in spatial abilities (reviewed by Hampson, 1995; Martin et al., 2007 for review), played a role in our observed results. Increased estrogen levels have been associated with decreases in visuospatial abilities (Gordon et al., 1986). In contrast, reduced levels of gonadotropin hormones, responsible for production of estrogen, are associated with superior visuospatial abilities (Gordon et al., 1986). Because the older females in our study were postmenopausal and not receiving estrogen hormone replacement, it is possible that the diminished estrogen levels in these women contributed to the lack of sex differences.

In the current study participants demonstrated a strong right-hand preference when provided with the opportunity to use both hands for grasping. This finding is consistent with previous research from our laboratory (Gonzalez et al., 2007; Stone et al., 2013) and the proposal of left hemisphere specialization for visually-guided actions (Goodale, 1988; Gonzalez et al., 2006, 2007; Serrien et al., 2006). Interestingly, right-hand use was differentially affected by the spatial complexity of the task, with right-hand use decreasing with increasing mental rotation demands. This finding is consistent, with the commonly held view (Corballis and Sergent, 1989; Ditunno and Mann, 1990) that mental rotation is primarily a right-hemisphere specialization. Although this result was seen in the overall ANOVA (YA and OA), it appeared to be more specific to the OA. Perhaps hand use in older adults is more malleable in response to task requirements in particular spatial demands. Further investigation is needed to ascertain if mental rotation and/or spatial visualization abilities influence hand use in both young and older adults.

Finally, is worth mentioning that although the task developed in this study possesses many commonalities to the standardized spatial tests, uniquely, it features the real-world interaction of reaching for, grasping, and assembling objects located in the environment. Each day, we are required to engage in touching and grasping of things around us. We must rely on these visuospatial abilities to be able to actively affect our surroundings. Because the developed task can be modified through manipulation of block size and model configuration the task is suitable for assessing visuospatial abilities in children (Sacrey et al., 2012), young, and older adults (Gonzalez et al., 2014) and likely pathological populations (e.g., people with Parkinson’s disease or people with visuospatial neglect, research in progress). Interestingly, research is increasingly suggesting that spatial abilities are malleable and can be trained. This flexibility presents the possibility of designing training or rehabilitation strategies which could be implemented to minimize identified disparities or impairments in spatial performance whether these differences are a consequence of sex, or other identified influences on spatial cognition such as socio-economic status (Levine et al., 2005; Hackman and Farah, 2009), aging (Klencklen et al., 2012), or neurological disorders (Vallar, 2007; Possin, 2010).

In conclusion, the present study developed a novel tool to assess visuospatial abilities. Older adults consistently performed the visuomotor task slower than the younger participants, however, their performance was comparable when expressing the results as a function of the task demands percent change. Importantly, because the visuomotor demands of the task were consistent between conditions, the difference in the time to complete the tasks resulted from the manipulation of visuospatial complexity. The presented task would be well suited to investigations of visuospatial function in the visuomotor domain, particularly with respect to sex and/or development and pathology.

## Conflict of Interest Statement

The authors declare that the research was conducted in the absence of any commercial or financial relationships that could be construed as a potential conflict of interest.
